# Impact of storage conditions and premix type on fat-soluble vitamin stability^1^

**DOI:** 10.1093/tas/txaa143

**Published:** 2020-08-08

**Authors:** Marut Saensukjaroenphon, Caitlin E Evans, Chad B Paulk, Jordan T Gebhardt, Jason C Woodworth, Charles R Stark, Jon R Bergstrom, Cassandra K Jones

**Affiliations:** 1 Department of Grain Sciences and Industry, College of Agriculture, Kansas State University, Manhattan, KS; 2 Department of Diagnostic Medicine/Pathobiology, College of Veterinary Medicine, Kansas State University, Manhattan, KS; 3 Department of Animal Sciences and Industry, Kansas State University, Manhattan, KS; 4 Animal Nutrition and Health, DSM Nutritional Products, North America, Parsippany, NJ

**Keywords:** fat-soluble vitamins, medium chain fatty acids, storage, vitamin stability

## Abstract

Feed ingredients and additives could be a potential medium for foreign animal disease entry into the United States. The feed industry has taken active steps to reduce the risk of pathogen entry through ingredients. Medium chain fatty acid (MCFA) and heat pulse treatment could be an opportunity to prevent pathogen contamination. The objective of experiment 1 was to determine the impact of 0, 30, 60, or 90 d storage time on fat-soluble vitamin stability when vitamin premix (VP) and vitamin trace mineral premix (VTM) were blended with 1% inclusion of MCFA (1:1:1 blend of C6:C8:C10) or mineral oil (MO) with different environmental conditions. Samples stored at room temperature (RT) (~22 °C) or in an environmentally controlled chamber set at 40 °C and 75% humidity, high-temperature high humidity (HTHH). The sample bags were pulled out at days 0, 30, 60 and 90 for RT condition and HTHH condition. The objective of experiment 2 was to determine the effect of heat pulse treatment and MCFA addition on fat-soluble vitamin stability with two premix types. A sample from each treatment was heated at 60 °C and 20% humidity. For experiment 1, the following effects were significant for vitamin A: premix type × storage condition (*P =* 0.031) and storage time × storage condition (*P =* 0.002) interactions; for vitamin D3: main effect of storage condition (*P <* 0.001) and storage time (*P =* 0.002); and for vitamin E: storage time × storage condition interaction (*P <* 0.001). For experiment 2, oil type did not affect the stability of fat-soluble vitamins (*P >* 0.732) except for vitamin A (*P =* 0.030). There were no differences for fat-soluble vitamin stability between VP and VTM (*P >* 0.074) except for vitamin E (*P =* 0.016). Therefore, the fat-soluble vitamins were stable when mixed with both vitamin and VTM and stored at 22 °C with 28.4% relative humidity (RH). When premixes were stored at 39.5 °C with 78.8%RH, the vitamin A and D_3_ were stable up to 30 d while the vitamin E was stable up to 60 d. In addition, MCFA did not influence fat-soluble vitamin degradation during storage up to 90 d and in the heat pulse process. The vitamin stability was decreased by 5% to 10% after the premixes was heated at 60 °C for approximately nine and a half hours. If both chemical treatment (MCFA) and heat pulse treatment have similar efficiency at neutralizing or reducing the target pathogen, the process of chemical treatment could become a more practical practice.

## INTRODUCTION

Vitamins are essential components for metabolism of protein, carbohydrates, and fat. Vitamin deficiencies could affect animal performance by decreasing growth rate or increasing the incidence of reproductive failures and osteoporosis (Mariana et al., 2019). There are many factors that can influence the stability of vitamins in premixes such as vitamin source, temperature, water content, pH, time, presence of choline, oxygen, light, and catalytic minerals (DSM Vitamin Nutrition Compendium, 2019). Typically, vitamin concentrations in complete feed are dependent on vitamins provided by the premix. These concentrations can be affected by storage conditions, storage time, and feed manufacturing process.

Pure vitamin production is limited to certain countries; therefore, they must be imported by a majority of countries, including the United States. Previous research has demonstrated that pathogenic viruses such as Porcine Epidemic Diarrhea Virus (PEDV) and African Swine Fever Virus (ASFV) can survive in certain feed ingredients and feed additives under simulated transport conditions (Dee et al., 2018). Therefore, precautionary steps need to be considered in order to reduce the risk of disease transmission through feed. Feed additives, temperature, and exposure time are options to consider. For instance, Cochrane et al., 2016 demonstrated that 1% of an MCFA blend effectively mitigated PEDV in feed ingredients. However, the negative effects the pathogen reducing procedures have on vitamin stability need to be determined. Therefore, the first objective of this experiment is to determine the impact of 0, 30, 60, or 90 d storage time on vitamin stability when stored as a vitamin premix (VP) or VTM and blended with 1% inclusion of MCFAs (1:1:1 blend of C6:C8:C10) or mineral oil (MO) with different environmental conditions. In addition, pathogens could be eliminated by a combination between temperature and exposure time. For instance, ASFV can be inactivated at 60 °C in 20 min (OIE 2009), while PEDV can reduce activity about 5.5 log when heated at 60 °C for 30 min (Hofmann and Wyler, 1989). Thus, heat pulse treatment could be an opportunity to prevent pathogen movement from a high-risk area to a clean area. However, the heat pulse treatment could denature or destroy vitamins. The second objective of this experiment is to determine the effect of heat pulse treatment and MCFA addition on vitamin stability with two premix types.

## MATERIAL AND METHOD

### Mixing Procedure

**Figure 1.**
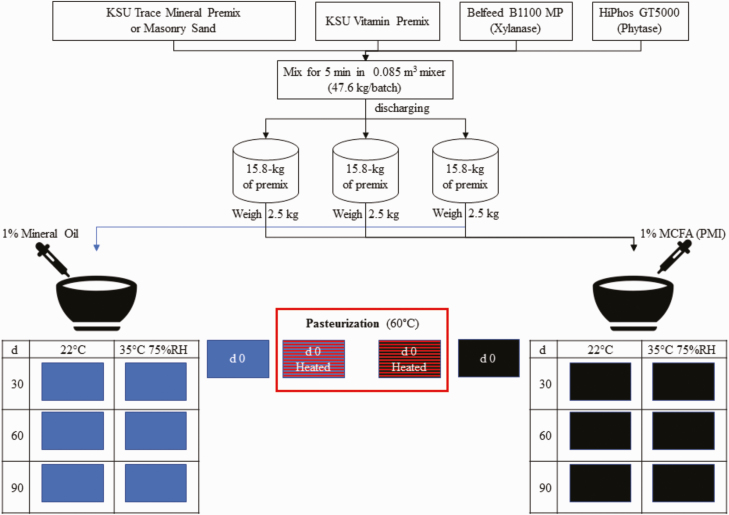
Flow chart of mixing steps used to create experimental treatments. Ingredients were mixed for 5 min in 47.6-kg batches using a 0.085 m^3^ paddle mixer (Davis model 2014197-SS-S1, Bonner Springs, KS). Then, each premix was equally discharged into three separate 15.9 kg aliquots. A 2.5-kg subsample of each aliquot was taken to create a 7.5-kg experimental premix treatment. The 7.5-kg premixes were mixed for 10 s using a mixer (Hobart model HL-200, Troy, OH). Following the 10 s dry mix, either a 74.8-g of 1:1:1 commercial blend of C6:0, C8:0 and C10:0 MCFA (PMI Nutritional Additives, Arden Hills, MN) or 74.8-g of MO were added using a pressurized hand-held sprayer with a fine hollow cone spray nozzle (UNIJET model TN-SS-2, Wheaton, IL). The premixes were mixed for an additional 90 s post oil application. The mixed samples were divided to obtain eight individual 900 g samples, which were placed in single-lined paper bags. Samples were then stored at RT in a temperature-controlled laboratory (~22 °C) or in an environmentally controlled chamber (Caron model 6030, Marietta, OH) set at 40 °C and 75% RH. In addition, separate samples were heated in an environmentally control chamber (Caron model 6030, Marietta, OH) at 60 °C and 20% RH.

A VP and a VTM were manufactured for both heat pulse treatment and storage condition experiments as outlined in Table 1. Both premixes contained phytase and phytase stability results are presented by Saensukjaroenphon et al. (2020). Masonry sand was added to the VP to keep the concentrations of the vitamins the same between the VP and VTM. Ingredients were mixed for 5 min in 47.6-kg batches using a 0.085 m^3^ paddle mixer (Davis model 2014197-SS-S1, Bonner Springs, KS). Then, each premix was equally discharged into three separate 15.9 kg aliquots. A 2.5-kg subsample of each aliquot was taken to create a 7.5-kg experimental premix sample. The 7.5-kg premixes were mixed for 10 s using a mixer (Hobart model HL-200, Troy, OH) equipped with an aluminum flat beater model HL-20 that had 3.69 % coefficient of variation when it was validated for uniform liquid addition. Following the 10 s dry mix, either a 74.8-g of 1:1:1 commercial blend of C6:0, C8:0, and C10:0 MCFA (PMI Nutritional Additives, Arden Hills, MN) or 74.8-g of MO were added using a pressurized hand-held sprayer with a fine hollow cone spray nozzle (UNIJET model TN-SS-2, Wheaton, IL). The premixes were mixed for an additional 90 s post oil application. The mixed samples were divided to obtain eight individual 900-g samples, which were placed in single-lined paper bags. These samples served as the experimental unit for all treatments. This process was repeated to yield three replicates per treatment. The mixing steps are illustrated in Figure 1.

**Table 1.** The composition of VP and VTM

	VTM		VP	
Ingredients	% Inclusion	Batch, kg	% Inclusion	Batch, kg
KSU swine vitamin^1^	25.89	57.07	54.35	25.89
KSU trace mineral^2^	32.60	15.53	0.00	0.00
Masonry sand	0.00	0.00	32.60	15.53
HiPhos GT5000^3^	8.70	4.14	8.70	4.14
Belfeed B 1100 MP^4^	4.35	2.07	4.35	2.07
Total	100.00	47.63	100.00	47.63

^1^Composition per kilogram: 1,653,000 IU vitamin A, 661,376 IU vitamin D3, 17,637 IU vitamin E, 13.3 mg vitamin B12, 1,323 mg menadione, 3307 mg riboflavin, 11,023 mg d-pantothenic acid, and 19,841 mg niacin. Rice hulls and calcium carbonate are carriers in the premix.

^2^Composition per kilogram: 73 g iron, 73 g zinc, 22 g manganese, 11 g copper, 198 g iodine, and 198 g selenium. Calcium carbonate is a carrier in the premix.

^3^Composition per kilogram: 5,000,000 FYT phytase (*Aspergillus oryzae*).

^4^Composition per kilogram: 98,000 U Xylanase (*Bacillus subtilis*).

### Storage Condition Experiment

Samples were stored at RT in a temperature-controlled laboratory (~22 °C) or in an environmentally controlled chamber (Caron model 6030, Marietta, OH) set at 40 °C and 75% humidity, high heat high humidity. The sample bags were pulled out at days 0, 30, 60, and 90 for RT condition and at days 30, 60, and 90 for high temperature and high humidity (HTHH) condition. The actual storage temperature and humidity for both conditions were collected using a data logger (HOBO model Onset U12-012, Bourne, MA). For the RT condition, the average temperature was 22.0, 22.1, and 22.1 °C; and the average relative humidity (RH) was 28.4%, 23.0%, and 33.7% for days 0–30, 31–60, and 61–90, respectively. For the HTHH condition, the average temperature was 39.5°, 39.5°, and 39.5°C; and the average relative humidity was 78.3%, 79.0, and 79.1% for days 0–30, 31–60, and 61–90, respectively. The individual premix samples were riffle divided twice to yield two 225-g sub-samples then they were sent to laboratories for vitamin A (AOAC 974.29.45.1.02), D_3_ (AOAC 2011.12) and E (AOAC 971.30). Previous research reported by Frye (1994) determined that the lower assay tolerance of vitamin E is 82%. Therefore, values ≥82% are not considered reportable in this experiment. The vitamin concentration at day 0, which was the initial concentration, was reported in international unit (IU) per kilogram. The results of vitamin at days 30, 60, and 90 were reported in percent stability, which was calculated by dividing the vitamin concentration by the initial vitamin concentration and then multiplying by 100.

### Heat Pulse Treatment Experiment

A sample from each treatment (2 × 2 factorial, with two premix types [VP or VTM] and two oil types [MO or MCFA]) was heated in an environmentally control chamber (Caron model 6030, Marietta, OH) at 60 °C and 20% humidity. The sample bags were pulled out after they were stored for 11 h and 48 min. The data logger (HOBO model Onset U12-012, Bourne, MA) was placed within the sample bag at approximately midlevel and remaining sample was placed on top to ensure data logger reflected true sample temperature. The premix temperature reached 60 °C after 2 h and 21 min in the chamber. The samples were held at 60 °C for 9 h and 27 min. The individual premix samples were riffle divided twice to yield two 225-g sub-samples, then they were sent to commercial laboratories for vitamin A (AOAC 974.29.45.1.02), D_3_ (AOAC 2011.12) and E (AOAC 971.30). The results of vitamin after heat pulse treatment were reported in percent stability, which was calculated by dividing the vitamin concentration by the initial vitamin concentration and then multiplying by 100.

### Statistical Analysis

Data were analyzed as two separate completely randomized experiments. The storage condition experiment, a sample storage bag was the experimental unit. Treatments were analyzed as a 2 × 2 × 2 × 4 factorial, with two premix type (VP or VTM), two oil type (MO or MCFA), two storage conditions (RT or HTHH), and three storage times (30, 60, or 90 d). The heat pulse treatment experiment, a mixing batch was the experimental unit. Treatments were analyzed as a 2 × 2 factorial, with two premix types (VP or VTM) and two oil types (MO or MCFA). Data were analyzed using the GLIMMIX procedure of SAS v9.4 (Cary, NC). Contrasts were used to compare the linear or quadratic effect of vitamin stability over time. Results were considered significant if *P* ≤ 0.05.

## RESULTS

### Initial Vitamin Concentrations

The initial concentration of vitamin A, D_3_, and E was reported in Table 2 for VP with MO, VP with MCFA, VTM with MO and VTM with MCFA. The formulated vitamin concentration was 898,406, 359,362, and 9,583 IU per kilogram for vitamin A, D_3_, and E, respectively. The initial concentration of three fat-soluble vitamins was more than 91% of formulated concentration for all four premixes.

**Table 2.** The analyzed fat-soluble vitamin concentrations of initial samples (day 0 and sampled immediately after mixing)

	VP		VTM	
Item	MO^1^	MCFA^2^	MO	MCFA
Vitamin A^1^, IU/kg	894,090	872,559	896,091	904,312
Vitamin D3^1^, IU/kg	339,652	349,823	341,702	328,184
Vitamin E^1^, IU/kg	9,032	9,250	9,620	9,584

^1^Included at 1% of the premixes; comprised of saturated aliphatic and alicyclic nonpolar hydrocarbons sourced as a by-product of petroleum refining.

^2^Included at 1% of the premixes; comprised of a 1:1:1 blend of MCFA (C6:0, C8:0, and C10:0) (PMI Nutritional Additives, Arden Hills, MN).

### Storage Condition Experiment

There were no four-way interactions among combinations of oil type, premix type, storage condition, and storage time (*P >* 0.200) for vitamin A. There was no evidence of an oil type × premix type × storage condition, oil type × storage condition × time or premix type × storage condition × time interaction (*P >* 0.332) for stability of vitamin A. There was a premix type × oil type × storage time interaction of vitamin A (*P =* 0.002; Table 3). Vitamin A was stable in VP mixed with MCFA and VTM mixed with MO when stored from 0 to 90 d. While increasing storage time continued to degrade vitamin A in VP mixed with MO and VTM mixed with MCFA. There was no evidence of an oil type × premix type, oil type × time, premix type × time interaction for stability of vitamin A (*P >* 0.051). There was a premix type × storage condition interaction (*P* < 0.01). When premixes were stored under HTHH, the VTM had higher vitamin A stability when compared with VP. However, there was no difference for vitamin A stability between VP and VTM when stored under RT. The oil type × storage condition interaction did not impact the fat-soluble vitamin stability (*P >* 0.339) except vitamin A (*P =* 0.009). The premixes with MO had a higher vitamin A stability compared with the premixes with MCFA when stored under RT. However, there was no difference for vitamin A stability between premix with MO and MCFA when stored under HTHH. There was a storage condition × time interaction (*P* < 0.01). When premixes were stored under HTHH, the vitamin A stability decreased as storage time increased to day 90. However, there was no difference in vitamin A stability as storage time increased to day 90 when stored under RT.

**Table 3.** Effect of the premix type, oil type, storage temperature, and storage time on vitamin A stability for storage condition samples

Item				
Premix type	Oil type^1^	Storage time, days	Storage condition	Vitamin A stability^6^, %
Interaction				
VP	MO^2^	30		91.8^bc^
VP	MO	60		88.5^c^
VP	MO	90		77.6^d^
VP	MCFA^3^	30		84.0^cd^
VP	MCFA	60		91.1^bc^
VP	MCFA	90		90.2^bc^
VTM	MO	30		98.9^ab^
VTM	MO	60		98.9^ab^
VTM	MO	90		92.8^bc^
VTM	MCFA	30		104.3^a^
VTM	MCFA	60		86.4^cd^
VTM	MCFA	90		83.8^cd^
Pooled SEM				3.4
VP			RT^4^	102.9^k^
VP			HTHH^5^	71.5^m^
VTM			RT	105.6^k^
VTM			HTHH	82.8^l^
Pooled SEM				2.0
	MO		RT	107.6^p^
	MCFA		RT	100.9^q^
	MO		HTHH	75.2^r^
	MCFA		HTHH	79.0^r^
	Pooled SEM			2.0
		30	RT	104.6^x^
		60	RT	104.3^x^
		90	RT	103.8^x^
		30	HTHH	84.9^y^
		60	HTHH	78.1^y^
		90	HTHH	68.4^z^
		Pooled SEM		2.4
Source of variation				
Oil type				0.453
Premix type				0.001
Oil type × premix type				0.051
Storage condition				<0.0001
Oil type × storage condition				0.009
Premix type × storage condition				0.031
Oil type × premix type × storage condition				0.679
Time				0.003
Oil type × time				0.382
Premix type × time				0.059
Oil type × premix type × time				0.002
Storage condition × time				0.008
Oil type × storage condition × time				0.332
Premix type × storage condition × time				0.349
Oil type × premix type × storage condition × time				0.121

^1^Included at 1% of the premixes.

^2^MO comprised of saturated aliphatic and alicyclic nonpolar hydrocarbons sourced as a by-product of petroleum refining.

^3^MCFA, comprised of a 1:1:1 blend of MCFA (C6:0, C8:0, and C10:0) (PMI Nutritional Additives, Arden Hills, MN).

^4^RT, the average temperature and relative humidity were 22.1 °C and 28.4%, respectively.

^5^High heat and high humidity, the average temperature, and relative humidity were 39.5 °C and 78.8%, respectively.

^6^Percent vitamin stability was calculated by dividing the vitamin activity at days 30, 60, or 90 by the analyzed initial vitamin activity and then multiplying by 100.

^a–d^Means within premix type × oil type × storage time interaction followed by a different letter are significantly different (*P ≤* 0.05)

^k–m^Means within premix type × storage condition interaction followed by a different letter are significantly different (*P ≤* 0.05)

^p–r^Means within oil type × storage condition interaction followed by a different letter are significantly different (*P ≤* 0.05)

^x–z^Means within storage condition × storage time interaction followed by a different letter are significantly different (*P ≤* 0.05)

There were no four-way, three-way, or two-way interactions among combinations of oil type, premix type, storage condition, and storage time (*P >* 0.073) for vitamin D_3_. There was no evidence of main effects (*P* > 0.424) of oil type or premix type on vitamin D stability. However, vitamin D_3_ stability was affected (*P <* 0.002) by the storage condition and time (Table 4). The premixes stored under RT had a higher vitamin D_3_ stability compared with the premixes stored under HTHH. There was a decrease in vitamin D_3_ stability as storage time increased (*P =* 0.002) from days 30 to 60; however, there was no further decrease from days 60 to 90.

**Table 4.** Effect of the premix type, oil type, storage temperature, and storage time on vitamin D_3_ stability for storage condition samples

Item				
Storage condition	Storage time, days	Premix type	Oil type^3^	Vitamin D_3_ stability^6^, %
Interaction				
RT^1^	30			92.3
RT	60			86.8
RT	90			89.4
HTHH^2^	30			87.8
HTHH	60			80.2
HTHH	90			77.0
Pooled SEM				2.0
Main effect				
RT				89.5^a^
HTHH				81.7^b^
Pooled SEM				1.2
	30			90.1^a^
	60			83.5^b^
	90			83.2^b^
	Pooled SEM			1.4
		VP		86.2
		VTM		84.9
		Pooled SEM		1.2
			MO^4^	85.8
			MCFA^5^	85.3
			Pooled SEM	1.2
Source of variation				
Oil type				0.752
Premix type				0.424
Oil type × premix type				0.781
Storage condition				<0.0001
Oil type × storage condition				0.339
Premix type × storage condition				0.721
Oil type × premix type × storage condition				0.793
Time				0.002
Oil type × time				0.465
Premix +				0.959
Oil type × premix type × time				0.676
Storage condition × time				0.141
Oil type × storage condition × time				0.421
Premix type × storage condition × time				0.282
Oil type × premix type × storage condition × time				0.073

^1^RT, the average temperature, and relative humidity were 22.1 °C and 28.4%, respectively.

^2^High heat and high humidity, the average temperature and relative humidity were 39.5 °C and 78.8%, respectively.

^3^Included at 1% of the premixes.

^4^MO comprised of saturated aliphatic and alicyclic nonpolar hydrocarbons sourced as a by-product of petroleum refining.

^5^MCFA, comprised of a 1:1:1 blend of MCFA (C6:0, C8:0, and C10:0) (PMI Nutritional Additives, Arden Hills, MN).

^6^Percent vitamin stability was calculated by dividing the vitamin activity at day 30, 60 or 90 by the analyzed initial vitamin activity and then multiplying by 100.

^a,b^Means within a main effect of storage condition followed by a different letter are significantly different (*P ≤* 0.05)

There were no four- or three-way interactions among combinations of oil type, premix type, storage condition, and storage time (*P >* 0.073) (Table 5) for vitamin E. There was no evidence of an oil type × storage condition or oil type × time interaction (*P >* 0.244) for stability of vitamin E. There were interactions (*P* < 0.016) for premix type × oil type, premix type × storage condition, and premix type × storage time for vitamin E stability. However, these interactions were not considered reportable because the percent stability of all treatments was 82% and above which was above the lower assay tolerance of vitamin E (82%) reported by Frye (1994). In addition, there was a storage condition × time interaction (*P* < 0.001) for vitamin E stability. Vitamin E was stable under both RT and HTHH up to 30 d. However, the degradation rate of vitamin E was faster when premixes were stored under HTHH vs. RT after 30 d of storage.

**Table 5.** Effect of the premix type, oil type, storage temperature, and storage time on vitamin E stability for storage condition samples

Item				
Storage condition	Storage time, days	Premix type	Oil type^3^	Vitamin E stability^6^, %
Interaction				
RT^1^	30			96.9^a^
RT	60			91.0^b^
RT	90			87.9^c^
HTHH^2^	30			96.0^a^
HTHH	60			83.9^d^
HTHH	90			79.6^e^
Pooled SEM				2.0
Main effect				
		VP		87.1^l^
		VTM		91.3^k^
		Pooled SEM		0.5
			MO^4^	88.1^y^
			MCFA^5^	90.4^x^
			Pooled SEM	0.5
Source of variation				
Oil type				0.002
Premix type				<0.0001
Oil type × premix type				0.016
Storage condition				<0.0001
Oil type × storage condition				0.542
Premix type × storage condition				<0.0001
Oil type × premix type × storage condition				0.200
Time				<0.0001
Oil type × time				0.244
Premix type × time				0.008
Oil type × premix type × time				0.609
Storage condition × time				<0.001
Oil type × storage condition × time				0.776
Premix type × storage condition × time				0.310
Oil type × premix type × storage condition × time				0.628

^1^RT, the average temperature and relative humidity were 22.1 °C and 28.4%, respectively.

^2^High heat and high humidity, the average temperature and relative humidity were 39.5 °C and 78.8%, respectively.

^3^Included at 1% of the premixes.

^4^MO comprised of saturated aliphatic and alicyclic nonpolar hydrocarbons sourced as a by-product of petroleum refining.

^5^MCFA, comprised of a 1:1:1 blend of MCFA (C6:0, C8:0, and C10:0) (PMI Nutritional Additives, Arden Hills, MN).

^6^Percent vitamin stability was calculated by dividing the vitamin activity at day 30, 60, or 90 by the analyzed initial vitamin activity and then multiplying by 100.

^a–d^Means within storage condition × storage time interaction followed by a different letter are significantly different (*P ≤* 0.05).

^k,l^Means within a main effect of premix type followed by a different letter are significantly different (*P ≤* 0.05).

^x,y^Means within a main effect of oil type followed by a different letter are significantly different (*P ≤* 0.05).

### Heat Pulse Treatment Experiment

There was no interaction between oil type and premix type (*P >* 0.287) for the stability of fat-soluble vitamins (Table 6). The oil type did not affect (*P >* 0.732) the stability of vitamins D_3_ and E. However, vitamin A stability was reduced (*P =* 0.030) in premixes containing MCFA after premixes were heated at 60 °C for 9 h and 27 min. The premix type did not affect (*P >* 0.074) the stability of vitamins A and D_3_. However, after the heat pulse treatment, vitamin E stability was reduced (*P =* 0.030) in VP compared with VTM.

**Table 6.** Effect of the premix type and oil type on vitamin stability of premix subjected to a pulse of high temperature (60 °C)

Item		Percent stability of^4^		
Premix type	Oil type^1^	Vitamin A	Vitamin D_3_	Vitamin E
Interaction				
VP	MO^2^	106.5	109.3	95.8
VP	MCFA^3^	90.4	105.7	94.6
VTM	MO	103.3	94.7	100.0
VTM	MCFA	97.0	99.4	100.0
	Pooled SEM	4.3	5.1	1.6
Main effect				
VP		98.4	107.5	95.2^y^
VTM		100.1	97.1	100.0^x^
Pooled SEM		3.0	3.6	1.1
	MO	104.9^a^	102.0	97.9
	MCFA	93.7^b^	102.6	97.3
	Pooled SEM	3.0	3.6	1.1
Source of variation				
Oil type × premix type		0.287	0.435	0.712
Oil type		0.030	0.911	0.732
Premix type		0.700	0.074	0.016

^1^Included at 1% of the premixes.

^2^MO comprised of saturated aliphatic and alicyclic nonpolar hydrocarbons sourced as a by-product of petroleum refining.

^3^MCFA, comprised of a 1:1:1 blend of MCFA (C6:0, C8:0, and C10:0) (PMI Nutritional Additives, Arden Hills, MN).

^4^Percent vitamin stability was calculated by dividing the vitamin activity at day 30, 60, or 90 by the analyzed initial vitamin activity and then multiplying by 100.

^a,b^Means within a main effect of oil type followed by a different letter are significantly different (*P ≤* 0.05).

^x,y^Means within a main effect of premix type followed by a different letter are significantly different (*P ≤* 0.05).

## DISCUSSION

### Storage Condition Experiment

Frye (1994) reported that the lower assay tolerance of fat-soluble vitamins was 85%, 86%, and 82% for vitamin A, D3, and E, respectively. After accounting for the variation of fat-soluble vitamin assays, the following effects remain significant for vitamin A: premix type × storage condition and storage time × storage condition interactions; for vitamin D3: main effect of storage condition and storage time; and for vitamin E: storage time × storage condition interaction.

Vitamin A was more stable when premixes were stored under RT regardless of oil type, and storage time compared with premixes that were stored under HTHH. Vitamin A continued to degrade when premixes were stored at HTHH longer than 30 d regardless of oil type. There was no reduction in vitamin A when premixes were stored under RT up to 90 d while the vitamin A stability decreased from 84.9% to 68.4 when premixes were stored under HTHH from 30 to 90 d regardless of oil type. Vitamin A was more stable in VTM (82.8%) vs. VP (71.5%), when premixes were stored under HTHH regardless of oil type and storage time. Gadient (1986) reported that vitamin A was highly sensitive to both temperature and oxygen and moderately sensitive to humidity. The result of the current study demonstrated the combination of temperature, high humidity and exposed time affected the vitamin A stability when premixes were stored at HTHH for 90 d (68.4%) which is in agreement with Gadient’s report.

The vitamin D_3_ stability was greater when premixes were stored in RT (89.5%) vs. HTHH (81.7%) regardless of oil type and storage time. However, when premixes were stored under HTHH for 30 d, the vitamin D_3_ stability was 87.8%. Increasing storage time from 30 to 90 d decreased the vitamin D_3_ stability from 90.1% to 83.2% regardless of premix type, oil type, and storage condition. The vitamin D_3_ stability was similar when premixes were mixed with MO (85.8%) vs. MCFA (85.3%) regardless of storage condition and storage time. Gadient (1986) reported that vitamin D_3_ was moderately sensitive to both temperature and humidity, and highly sensitive to oxygen. The result of the current study demonstrated that the combination of temperature and high humidity affected the vitamin D_3_ stability when premixes were stored at HTHH regardless of oil type.

Vitamin E was more stable when premixes were stored longer than 30 d under RT vs. HTHH regardless of oil type. The vitamin E stability was similar when premixes were stored shorter than 30 d under RT (96.9%) vs. HTHH (96.0%) regardless of oil type. The vitamin E stability was above 88% when premixes were stored under RT up to 90 d while the vitamin E stability decreased from 96% to 80% when premixes were stored under HTHH from 30 to 90 days regardless of oil type. The premixes were mixed with MCFA (90.4%) had a higher vitamin E stability compared with the premixes that were mixed with MO (88.1%) regardless of storage condition and storage time. Gadient (1986) reported that vitamin E was slightly sensitive to both temperature and humidity, and moderately sensitive to oxygen. The result of the current study demonstrated that the combination of temperature, high humidity, and exposed time affected the vitamin E stability when premixes were stored at HTHH for 90 d (79.6%) regardless of oil type which in agreement with Gadient’s report.

The water molecules, oxygen in the air and temperature may influence the oxidation rate of fat-soluble vitamins; therefore, resulting in decreased vitamin stability when premixes were stored under 39.5°C and 78.8% relative humidity. This is supported by Tavcar-Kalcher and Vengust (2007) who reported that the oxidation of some vitamins was catalyzed by air, light, heat, moisture, mineral acids, metal ions, unsaturated fats, and oxidants. The MCFA did not affect the stability of fat-soluble vitamins.

### Heat Pulse Treatment Experiment

Gadient (1986) reported that the heat sensitivity was highly, moderately, and slightly for vitamins A, D_3_, and E, respectively. Additionally, the vitamin A stability was 87% when the feed was steam-conditioned at 60 °C and then pelleted. The current study indicated that when premixes with either MO or MCFA were heated at 60 °C for 9 h and 27 min, the vitamin stability was more than 90%, 94.7%, and 94.6% for vitamin A, D3, and E, respectively, which was in agreement with the results of Gadient’s study. In addition, the result of the current study demonstrated that the stability of vitamin D_3_ and E was similar when premixes were mixed with MO or MCFA. The vitamin A stability was higher when premixes contained MO (104.9%) vs. MCFA (93.7%). However, vitamin A stability was still >90%, and it is hypothesized that the differences were caused by laboratory variation. The degradation of fat-soluble vitamins was between 5% and 10% after heat pulse treatment.

## CONCLUSION

The fat-soluble vitamins were stable when mixed with both vitamin and VTM and stored at 22 °C with 28.4%RH. When premixes were stored at 39.5 °C with 78.8%RH, the vitamins A and D_3_ were stable up to 30 d while the vitamin E was stable up to 60 d. In addition, MCFA did not negatively affect fat-soluble vitamin degradation during storage up to 90 d and in the heat pulse process. The vitamin stability was >90% after the premixes were heated at 60 °C for approximately nine and a half hours. If both chemical treatment (MCFA) and heat pulse treatment have similar efficiency at neutralizing or reducing the target pathogen, the process of chemical treatment could become a more practical practice.



*Conflict of interest statement*. Jon R. Bergstrom is employed by DSM Nutritional Products which provided partial support for the experiment. The authors have no additional real or perceived conflicts of interest to declare.
